# Aglycone specificity of *Thermotoga neapolitana *β-glucosidase 1A modified by mutagenesis, leading to increased catalytic efficiency in quercetin-3-glucoside hydrolysis

**DOI:** 10.1186/1471-2091-12-11

**Published:** 2011-02-23

**Authors:** Samiullah Khan, Tania Pozzo, Márton Megyeri, Sofia Lindahl, Anders Sundin, Charlotta Turner, Eva Nordberg Karlsson

**Affiliations:** 1 Biotechnology, Dept of Chemistry, Lund University, P.O. Box 124, SE-221 00 Lund, Sweden; 2 Institute of Enzymology, Hungarian Academy of Sciences, H-1113 Budapest, Karolina út 29, Hungary; 3 Organic Chemistry, Lund University, P.O. Box 124, SE-221 00 Lund, Sweden

## Abstract

**Background:**

The thermostable β-glucosidase (*Tn*Bgl1A) from *Thermotoga neapolitana *is a promising biocatalyst for hydrolysis of glucosylated flavonoids and can be coupled to extraction methods using pressurized hot water. Hydrolysis has however been shown to be dependent on the position of the glucosylation on the flavonoid, and e.g. quercetin-3-glucoside (Q3) was hydrolysed slowly. A set of mutants of *Tn*Bgl1A were thus created to analyse the influence on the kinetic parameters using the model substrate *para*-nitrophenyl-β-D-glucopyranoside (*p*NPGlc), and screened for hydrolysis of Q3.

**Results:**

Structural analysis pinpointed an area in the active site pocket with non-conserved residues between specificity groups in glycoside hydrolase family 1 (GH1). Three residues in this area located on β-strand 5 (F219, N221, and G222) close to sugar binding sub-site +2 were selected for mutagenesis and amplified in a protocol that introduced a few spontaneous mutations. Eight mutants (four triple: F219L/P165L/M278I, N221S/P165L/M278I, G222Q/P165L/M278I, G222Q/V203M/K214R, two double: F219L/K214R, N221S/P342L and two single: G222M and N221S) were produced in *E. coli*, and purified to apparent homogeneity. Thermostability, measured as T_m _by differential scanning calorimetry (101.9°C for *wt*), was kept in the mutated variants and significant decrease (ΔT of 5 - 10°C) was only observed for the triple mutants. The exchanged residue(s) in the respective mutant resulted in variations in K_M _and turnover. The K_M_-value was only changed in variants mutated at position 221 (N221S) and was in all cases monitored as a 2-3 × increase for *p*NPGlc, while the K_M _decreased a corresponding extent for Q3.

Turnover was only significantly changed using *p*NPGlc, and was decreased 2-3 × in variants mutated at position 222, while the single, double and triple mutated variants carrying a mutation at position 221 (N221S) increased turnover up to 3.5 × compared to the wild type. Modelling showed that the mutation at position 221, may alter the position of N291 resulting in increased hydrogen bonding of Q3 (at a position corresponding to the +1 subsite) which may explain the decrease in K_M _for this substrate.

**Conclusion:**

These results show that residues at the +2 subsite are interesting targets for mutagenesis and mutations at these positions can directly or indirectly affect both K_M _and turnover. An affinity change, leading to a decreased K_M_, can be explained by an altered position of N291, while the changes in turnover are more difficult to explain and may be the result of smaller conformational changes in the active site.

## Background

Glycoside hydrolases (GH) are enzymes that hydrolyse glycosidic bonds between two or more carbohydrates, or between a carbohydrate and a non-carbohydrate moiety. Carbohydrates are essential components of biomass, which is estimated to be produced in a quantity of about 60 Gt/year [1] and contain an array of structural and storage polysaccharides. To utilize these raw materials, microorganisms produce a wide variety of carbohydrate hydrolysing and modifying glycoside hydrolases. These enzymes can also be used as specific catalysts in industrial applications, *e.g*. in the food and feed industries, the paper and pulp, starch and textile industries, and in newly emerging sustainable processes [2,3] taking advantage of their specificity in selective preparations of carbohydrate-containing raw materials.

Antioxidants are bioactive compounds that have received great interest due to their potential as health beneficial agents. The action of antioxidants is to counteract oxidative stress imposed by reactive oxygen species shown to play a crucial role in the pathophysiology associated with neoplasia, atherosclerosis and neurodegenerative diseases [4,5]. Polyphenolic compounds show a wide range of antioxidant activities, and are thought to exert protective effects against the diseases specified above [5-8]. Flavonoids are polyphenolic compounds that are important antioxidative constituents of fruits and vegetables, but the type of compound is different in different sources. Fruits and vegetables rich in anthocyanins (*e.g*. strawberry, raspberry and red plum) show highest antioxidant activities, followed by those rich in flavonones (*e.g*. orange and grapefruit) or flavonols (*e.g*. onion, leek, spinach and green cabbage), while hydroxycinnamate-rich fruits (*e.g*. apple, tomato, pear and peach) exhibit lower antioxidant activities [5,9]. Flavonols are generally not found as free aglycones (*e.g*. quercetin and kaempferol), but rather as complex conjugates with sugar residues (*e.g*. glucose or rhamnose). GHs hydrolyse certain flavonoid glycosides, dependent on the aglycone moiety, type of sugar and linkage [3,10] and some glucosidases classified under GH family 1 (GH1) [11] are flavonoid-hydrolysing enzymes. In a previous work we have shown that the oligosaccharide hydrolysing GH1 β-glucosidase *Tn*Bgl1A, from the hyperthermophile *Thermotoga neapolitana*, efficiently hydrolyses quercetin-4'-glucosides (Q4') [3]. Q4' and quercetin-3,4'-diglucoside (Q3,4') are the most abundant species in yellow onion [12], and to obtain the quercetin aglycone both Q4' and Q3 species must be hydrolysed.

Most commonly quercetin and its glycosides have been extracted from yellow onion by simple liquid/solid extraction techniques (*e.g*. aqueous methanol) combined with chemically (*e.g*. HCl) catalyzed hydrolysis reaction of the extracted quercetin glucosides [12-16].

We have instead utilized pressurized hot water to extract the quercetin species from yellow onion, followed by biocatalytic conversion of the quercetin glucosides to quercetin and carbohydrates [3]. In this system, use of enzymes with high thermostability is essential, and the enzyme *Tn*Bgl1A from the hyperthermophile *T. neapolitana *is from this perspective a suitable biocatalyst [3,17]. It was also shown that the Q4' was much more efficiently hydrolysed than the Q3.

In this investigation, the β-glucosidase *Tn*Bgl1A, was mutated to investigate the influence of mutations on the enzyme kinetics (using the substrate *para-*nitrophenyl-β-D-glucopyranoside (*p*NPGlc)), thermostability, and selective hydrolysis of glucose at two positions (4' and 3) on the aglycone quercetin. We have utilized a strategy to design mutants based on bioinformatics and structural analysis, with an amplification protocol that allowed spontaneous mutations, in order to find residues that influence specificity of the enzyme.

## Methods

### Chemicals

All chemicals were of pro-analysis grade from Merck Eurolabs (Darmstadt, Germany) unless otherwise stated.

### Cloning of *Tnbgl1A*

The gene encoding Bgl1A was PCR-amplified from genomic *Thermotoga neapolitana *(DSM strain 4359) DNA as described by Turner et al [3]. Primers (1 and 2 with restrictions sites for cloning, *Nde*I and *Xho*I, underlined, Table 1) were designed to amplify the coding sequence of *bgl1A (*previously termed *gghA *[18]) from the sequence deposited at the NCBI server [19] under the accession number AF039487. The gene was inserted in vector pET-22b(+) (Novagen, Madison, WI, USA) under control of the T7/lac promoter and incorporating the C-terminal hexa-histidine tag [3]. The resulting plasmids were transformed into *E. coli *Nova Blue cells (Novagen) and screened by colony PCR using the T7 forward and T7 reverse primers (3 and 4, Table 1) and *Taq *DNA polymerase. Positive clones were transformed into the *E. coli *expression host Tuner (DE3) (Novagen). The complete gene was sequenced at MWG Biotech (Ebersberg, Germany).

**Table 1:** Oligonucleotides used for cloning and mutagenesis

Primer	Direction	Mutation	Sequence^a, b^
1	Forward	N/A	5'-TATTCTTATCATATGAAAAAGTTTCCCGAAGGGTTC
2	Reverse	N/A	5'-TATTCTTATCTCGAGATCTGTTAGTCCGTTGTTTTTG
3	Forward	N/A	5'-AATACGACTCACTATAGG
4	Reverse	N/A	5'-CTAGTTATTGCTCAGCGG
5	Forward	F219L	5'GACGGAAAGATAGGGATTGTT**TTA**AACAACGGATACTTCGA
6	Forward	N221S	5'-GGGATTGTTTTCAAC**AGC**GGATACTTCGAACCTGC
7	Forward	G222M	5'-GATTGTTTTCAACAAC**ATG**TACTTCGAACCTGCAAG
8	Forward	G222Q	5'- GGATTGTTTTCAACAAC**CAA**TACTTCGAACCTGCAAGTGAGAGAG
9	Reverse	F219L	5'- GACGGAAAGATAGGGATTGTT**TTA**AACAACGGATACTTCGA
10	Reverse	N221S	5'- GCAGGTTCGAAGTATCC**GCT**GTTGAAAACAATCCC
11	Reverse	G222M	5'-CTCTCTCACTTGCAG**TAC**CGAAGTATTGGTTGTTGAA
12	Reverse	G222Q	5'- CTCTCTCACTTGCAG**GTT**CGAAGTATTGGTTGTTGAAAACAATCC

^a^The restriction sites for cloning are underlined. ^b^Induced changes are shown in bold.

### Mutagenesis

Mutagenesis was performed in order to introduce the following designed changes: F219L, N221S, and G222M/Q, respectively. *Taq *polymerase, which lacks proofreading, (Invitrogen Life Technologies) was used (with wild-type gene as template) to allow introduction of a few random additional mutations. Standard concentrations of MgCl_2 _(1.5 mM) and dNTPs (200 μM) were used. In a first PCR (94°C 3 min; 35 cycles: 94°C 45 s, 55°C 30 s, 72°C 90 s, 72°C 10 min), a mutated gene fragment encoding the C-terminal part of the enzyme was constructed using forward mismatched primers (primers 5-8, mismatch in bold, Table 1) together with the reverse gene specific primer 2, encoding the C-terminal sequence of Bgl1A.

The N221S mutants were obtained by amplifying the full length gene in a second PCR (94°C 3 min; 35 cycles: 94°C 45 s, 60°C 30 s, 72°C 90 s, 72°C 10 min) using the product of the first PCR as a reverse "megaprimer" together with the forward primer 1 matching the start of the gene.

Mutations at position 219 (F219L), and 222 (G222M/Q) were constructed using overlap extension PCR of the mutated fragment and an overlapping gene fragment encoding the N-terminal part of Bgl1A. Reverse primers (9-12, Table 1) together with the forward gene specific primer 1 were used to create the overlapping fragments that were PCR-amplified (94°C, 3 min; 35 cycles: 94°C 45 s, 55°C 30 s, 72°C 90 s, 72°C 10 min) with the wild-type gene as a template. Overlap extension PCR reaction was then run in two steps. Firstly, extension without template at: 94°C 4 min; 10 cycles: 94°C 1 min, 47.5°C 1 min, 72°C 90 s, 72°C 7 min and the ramp between the annealing and extension changed from the default 3°C/s to 5°C/s. Secondly, amplification at standard conditions, using the gene-specific forward and reverse primers (1 and 2), and the product of the overlap extension PCR as template.

All mutated genes (inserts) were purified with QIAEXII Gel Extraction kit (Qiagen) after gel separation. Both insert and vector were digested and ligated as described under the cloning section. Resulting plasmids were transformed into *E. coli *Nova Blue cells (Novagen) and screened by colony PCR using the T7 forward and T7 reverse primers (3 and 4, Table 1). Selected mutant clones were fully sequenced at MWG Biotech.

### Homology modelling and ligand binding

A *Tn*Bgl1A homology model was constructed utilizing the Schrodinger 2010 software suite [20]. Energy minimizations were performed with Macromodel, utilizing the OPLS-2005 force field and the GB/SA model for water solvation. Molecular dynamics was performed with Desmond, utilizing default settings.

To find homologes to the *Tn*Bgl1A sequence, a BLAST search was performed on proteins with X-ray diffraction data in the PDB data base. The amino acid sequence of the GH1 β-glucosidase from *Thermotoga maritima *provided the highest similarity, 90% identity, and 97% positives. The PDB structure 2WC4 of BglA from *T. maritima *with 3-imino-2-thio-(+)-castanospermine as ligand, had the highest resolution of these X-ray crystallographic models (1.7 Å), and was used as template to build a 3D model of *Tn*Bgl1A (Prime version 2.2.108). The Protein Preparation Wizard was utilized to add hydrogens, assign charges, optimize hydrogen bond networks, and to analyze the quality of the homology model. The RMSD (over all α-carbons) with the template was 0.2 Å after optimisation.

In the region of the active site, a cis-bond between W396 and S397 was found, however this cis-amide is present in all crystals of *T. maritima *β-glucosidase. Furthermore, energy minimization showed the cis conformer to have lower energy than the corresponding trans amide bond.

A low energy model of β-D-cellotetraose was constructed [21] and placed in the homology model so that the β-D-glucose at the non-reducing end superimposed with the six membered rings of the *T. maritima *BglA ligands in the pdb structures 2WC4, 3CMJ, 1QOX, 1E4I, and 1BGA. The glycosidic linkage torsion angle ϕ of this sugar was adjusted from -121° to 26° to make cellotetraose fit in the ligand binding pocket. The resulting structure was energy minimized to yield a cellotetraose binding pose that is in agreement with the proposed mechanism of family 1 β-glucosidases [22]. The binding pose was verified as stable in 2 ns molecular dynamics simulation.

A conformational search was performed on Q3, and the lowest energy conformer was placed in the homology model using the same method as for cellotetraose. The resulting structure was energy minimized, and the binding pose, which was in agreement with the proposed mechanism of family 1 β-glucosidases, was verified with a 2 ns molecular dynamics simulation.

Figures of docking results were prepared within the Schrodinger 2010 software suite, including the program PyMol.

### Expression and purification

The mutant and wild-type enzymes were produced in 2.5 L batch cultivations at 37°C, pH 7, using a defined medium [23] with 100 μg mL^-1 ^ampicillin and a dissolved oxygen tension (DOT) above 40%. Expression was induced at OD_620 nm _= 3, by the addition of 0.1-1 mM isopropyl-beta-D-thiogalactopyranoside (IPTG), and continued for 3 h. Production levels were analysed by SDS-PAGE (see below) in samples (1 mL) withdrawn hourly after induction. The cells were pelleted, resuspended in 300 μL 50 mM citrate-phosphate buffer, pH 5.6, ultrasonicated for 2 × 90 s with a UP400S equipped with a 3 mm titanium probe (Dr. Hielscher, Stahnsdorf, Germany) using a sound intensity of 60% and a cycle of 0.5 and thereafter centrifuged for 15 min at 13 000 × *g *to separate soluble proteins from insoluble proteins and cell debris.

The cells were harvested, separated from the cultivation medium by centrifugation (10000 × *g*, 10 min, 4°C), and dissolved in binding buffer (20 mM imidazole, 20 mM Tris-HCl, 0.75 M NaCl, pH 7.5). The ice-chilled cell suspension was lysed by sonication for 5 × 3 min using a 14 mm titanium probe sound intensity of 60% and a cycle of 0.5 (UP400 S, Dr. Hielscher), centrifuged (30 min, 39000 × *g*, 4°C), heat treated (70°C, 30 min) and again centrifuged. The supernatant was passed through a 0.45 μm Minisart high-flow filter (Sartorius, Göttingen, Germany) and purified on an ÄKTA prime system (Amersham Biosciences, Uppsala, Sweden) by immobilized metal ion affinity chromatography using copper as a ligand as described elsewhere [24]. The fractions containing the purified protein were pooled and dialyzed against 20 mM citrate phosphate buffer, pH 5.6, overnight using a Spectra/Por dialysis membrane with a 3500 Da molecular weigh cut-off (Spectrum laboratories, Rancho Dominguez, CA, USA). The dialysed protein fractions were stored at 4°C until use.

### Protein analysis

The purity of each mutant and wild-type enzyme was analysed by SDS-PAGE according to [25]. Expression levels were also analysed by SDS-PAGE after separating insoluble and soluble proteins.

Total protein concentration was estimated at 562 nm by the BCA method (Sigma, Steinheim, Germany) using bovine serum albumin (Sigma-Aldrich) as standard.

### Differential scanning calorimetry (DSC)

DSC analysis was made on a MicroCal differential scanning calorimeter (VP-DSC, MicroCal, Northampton, MA, USA) with the cell volume of 0.5072 mL. The samples (in 20 mM citrate phosphate buffer, pH 5.6) were concentrated to 1 mg mL^-1 ^using Vivaspin (Sartorius AG, Goettingen, Germany) centrifuging tubes with a MWCO of 30,000 Da and were degassed before the scans. The samples were scanned at a rate of 1°C/min in the temperature range of 25-110°C.

### Enzyme activity on *p*NPGlc, Q3 and Q4'

**Figure 1:**
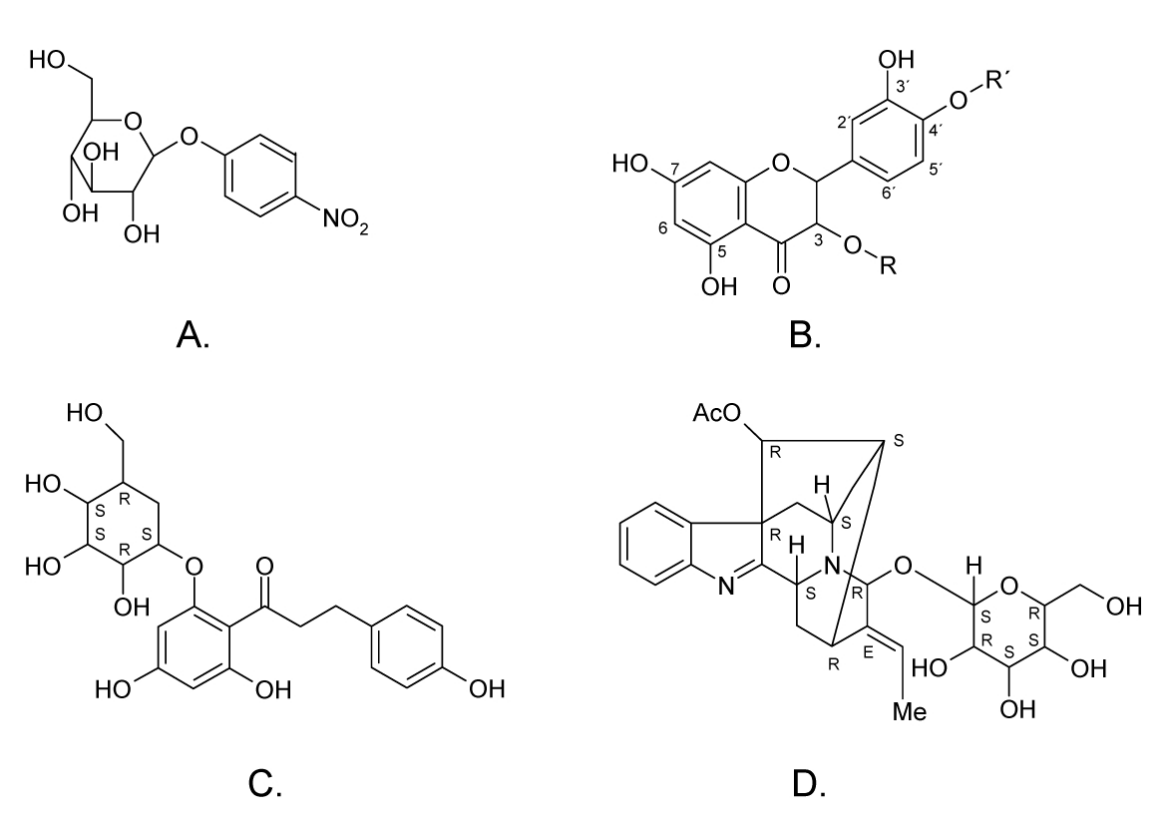
**Schematic structures of the substrates used for enzyme activity analysis and the bulky alkaloid substrates hydrolysed by some GH1 representatives**. Structures of *para*-nitrophenyl-beta-D-glucopyranoside (**A**.), quercetin species (**B**.), and the alkaloids strictosidine (**C**.), and raucaffricine (**D**.). Quercetin (R, R' =  H), quercetin-3-glucoside (R = glucose and R' = H), quercetin-4'-glucoside (R = H and R' = glucose) and quercetin-3,4'-glucoside (R, R' = glucose).

Enzyme activity, and kinetic parameters (K_M _and k_cat_) were determined at 80°C, pH 5.6 using *p*NPGlc (*para*-nitrophenyl-β-D-glucopyranoside, Figure 1A) as substrate in 20 mM citrate phosphate buffer, on a Shimadzu UV-1650 Visible spectrophotometer (Shimadzu, Duisburg, Germany). A volume of 980 μL of *p*NPGlc (in a concentration range from 0.09125 to 1 mM) was preheated for 10 min, where after 20 μL of the enzyme solution (12 μg mL^-1^, 4.56 pmol) was added. Absorbance at 405 nm was measured and plotted by the Shimadzu UV probe 2.01 software as a function of time during 1 min. The extinction coefficient of *p*NP (*para*-nitrophenol) under these experimental conditions was determined as ε _80°C, 405 nm_= 2.4639 × 10^3 ^mL mmol^-1^cm^-1^. The kinetic parameters were determined by applying the Wilkinson non-linear regression method using Enzpack (Biosoft, UK). The effect of glucose on activity in this system was evaluated by adding 10 mM glucose to the stock solution of 1 mM *p*NPGlc and the kinetic values K_M _and k_cat _were determined as above.

Enzyme activity of selected mutants of *Tn*Bgl1A were screened for two quercetin glucosides (Figure 1B), quercetin-3-glucoside (Q3) (Polyphenols Laboratories AB, Sandnes, Norway) and quercetin-4'-glucoside (Q4') (Polyphenols Laboratories AB) at 90°C. 200 nmol samples of Q3 or Q4' dissolved in methanol (in triplicate) were evaporated and 1.0 mL of 100 mM citrate phosphate buffer, pH 5.0, was added and the vials were heated at 90°C until substrate was dissolved. A 50 μL fraction was collected and added to 450 μL of mobile phase composed of methanol/water (50:50) and 0.13 M formic acid. The reaction was started by adding 20 pmol of enzyme and 5 min after addition of enzyme, 50 μL fractions were collected and added to 450 μL of mobile phase. Samples were analyzed by HPLC with UV detection (HPLC-UV). The conditions and methodolology for the kinetic measurement of Q3 and Q4' was published in Lindahl et al [17]. In summary, 33-167 nmol Q3 and Q4' were dissolved per ml citrate-phosphate buffer pH 5.0. For Q3 hydrolysis 200 pmol *wt *and 20 pmol mutant N221S/P342L were used, and for Q4' hydrolysis 20 pmol *wt *and 10 pmol mutant N221S/P342L were used.

### HPLC analysis

HPLC-UV analysis was performed using the chromatographic system UltiMate 3000 from Dionex (Germering, Germany). An Agilent Zorbax SB-C18 column (100 × 2.1 mm, 3.5 μm) was used for isocratic separation with a methanol:water (50:50) and 0.13 M formic acid mobile phase at a flow rate of 0.15 mL min^-1^. The injection volume was 10 μL and detection was accomplished at 350 nm. Quantification of quercetin and glycosides was performed using a five-point calibration curve of a quercetin dihydrate standard (Sigma-Aldrich, Steinheim, Germany) and Q3 and Q4' standards at concentrations between 0.5 and 25 μg mL^-1^. Each vial taken to analysis had a total volume of 500 μL.

## Results

The different members of glycoside hydrolase family 1 (GH1), catalyse hydrolysis of a glucose molecule from a number of different substrates, including some hydrophobic substrates linked to glucose. Only a few members of the family are, however, commercially available, and many different analytical assays have thus utilized β-glucosidase from almond, which has been classified under GH1 [26], and which is often available in heterogeneous preparations. β-Glucosidase A from *Thermotoga neapolitana*, *Tn*Bgl1A, was chosen for this work as an interesting candidate based on previous promising results in biocatalytic conversion of quercetin glucosides to quercetin and carbohydrates in yellow onion extract, extracted using a pressurized hot water extraction technique [3]. This enzyme is thermostable, and hence suitable for application in hot water. Moreover, this enzyme belongs to a GH family with diverse substrate specificity (including enzymes active on both oligosaccharides and larger substrates), with many gene sequences available allowing comparison, and with necessary structural information available, including three-dimensional (3D) structures of the closely related enzyme *Tm*GH1 from *Thermotoga maritima *[27,28], allowing homology modelling of the 3D structure.

### Structural considerations and mutation strategy

As noted elsewhere [3], the deduced amino acid sequence encoded by the *bgl1a*-gene used in this work has one change in primary sequence (G436V) compared to the deposited sequence (NCBI accession number AAB95492). Sequence alignments revealed V at position 436 to be conserved among several members of GH family 1, and the obtained sequence is hereafter referred to as wild type (*wt*) and designated *Tn*Bgl1A.

**Figure 2:**
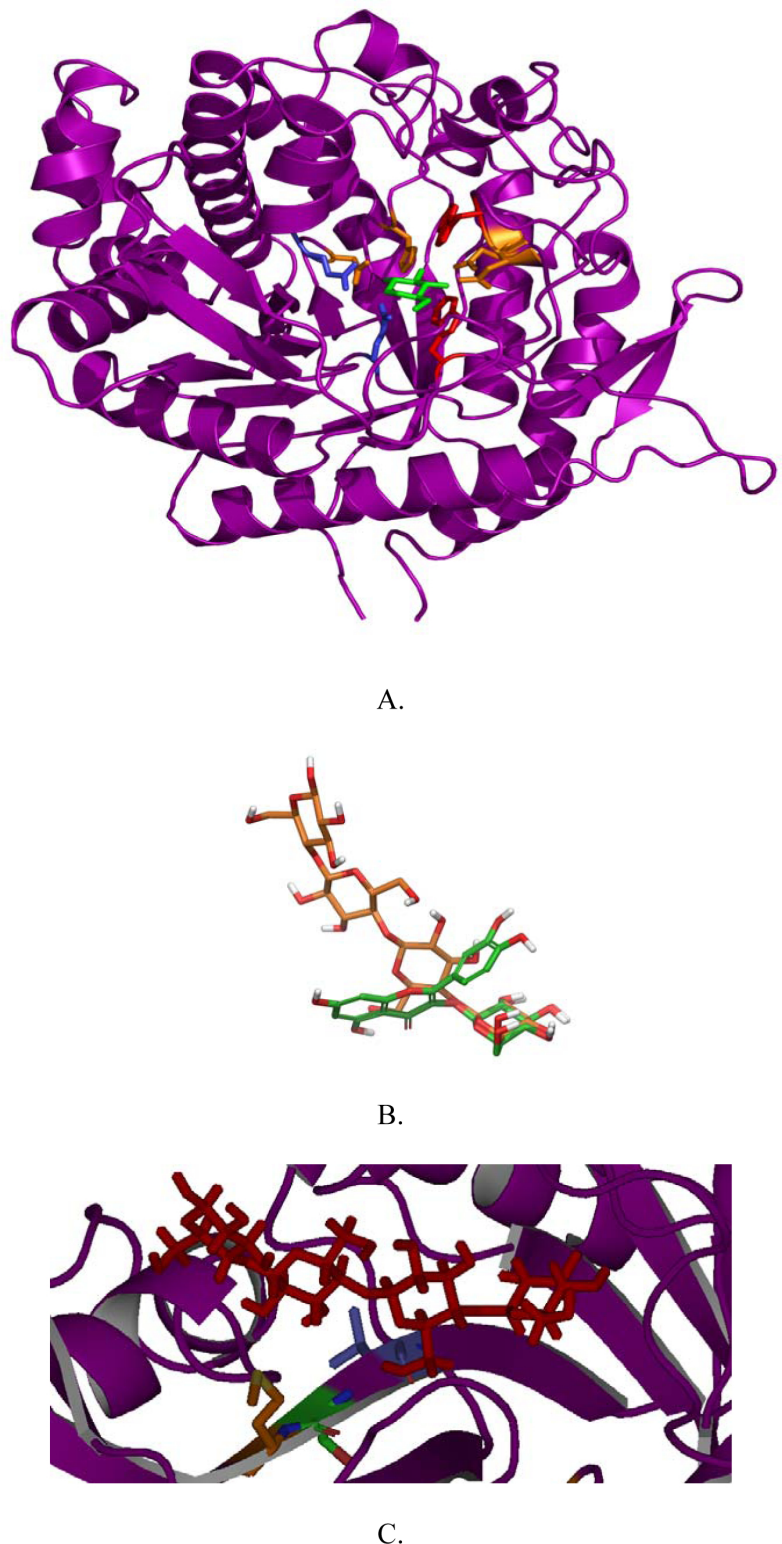
**Overall structure of *Tn*Bgl1A, relative substrate positions and the cellotetraose position in relation to mutated residues**. *Tn*Bgl1A homology model showed the typical (β/α)_8 _barrel fold (**A**.), a feature of the overall structure in GH1. The proton donor E164 and nucleophile E349 of the enzyme are illustrated in blue and shown in stick representation. In green the G2F inhibitor is shown in the -1 subsite, interacting with four residues in orange: (Q18, H119, N163, E403) by hydrogen bonds and two by hydrophobic interactions (W396, W404). In panel **B **the relative positions of the ligands cellotetraose (orange) and quercetin-3-glucoside (Q3, in green) when bound in the enzyme are shown. The matching binding of the glucopyranoside in the two substrates at the -1 subsite is shown to the right. The cellotetraose labelling from left to right correspond to subsites +3, +2, +1, -1. In panel **C **the cellotetraose (again positioned with the +3 subsite to the left), is displayed in the active site channel, and the selected residues close to the +2 subsite (from left to right:G222, N221 and F219) are shown in the mutated forms as M222, S221 and L219. The G222M was made to increase hydrophobicity at the entrance of the active site, while the G222Q mutation (not shown) was predicted to result in hydrogen bonding with the substrate. The F219L and N221S mutations were predicted to generate space for better substrate accommodation.

A molecular model of *T. neapolitana Tn*Bgl1A was generated based on the 3D structure of β-glucosidase BglA from *T. maritima *(here termed *Tm*GH1) [27,28]. *Tm*GH1 provided the highest similarity: 90% identity, and 97% positives. The homology detection structure prediction server HHpred [29,30] also confirmed *Tm*GH1 as the best template. The overall structure presented in the *Tn*Bgl1A model is the typical (β/α)_8 _barrel fold characteristic of GH1 (Figure 2A). The active site is a deep channel (18-21Å) narrowed at the bottom with a wider cleft at the entrance. Two conservative motifs TLN**E**P and IT**E**NG are situated opposite to each other inside the active site, more precisely at the ends of strands β4 and β7 containing the acid/base (E164) and the nucleophile (E349) residues respectively.

Superimposition of *Tm*GH1 in complex with 2-deoxy-2-fluoro-β-D-glucopyranoside (2GF) (PDB 1OIN) and *Tn*Bgl1A allowed identification of the residues forming the glycone binding site (or -1 subsite) (Figure 2A). Interacting residues included conservative residues forming hydrogen bonds (Q18, H119, N163, E403) and hydrophobic interactions (W396, W404).

Cellotetraose was modelled into the *Tn*Bgl1A model to analyse putative interactions at the +1 and +2 subsites. The β-D-glucopyranoside at the non-reducing end (-1 subsite) was placed by superimposing the six membered rings of the ligand (hexose rings or inhibitors) in 5 GH1 structures (see materials and methods for details). The sugars in the cellotetraose were positioned in a dynamically stable position in agreement with the proposed mechanism of family 1 β-glucosidases [22]. The same procedure was repeated for the glucosylated quercetin to see its position in relation to the sugar binding subsites. Both Q3 (Figure 2B) and Q4' could be fitted in positions relevant for hydrolysis, while the double glucoside (Q3,4') could not be accommodated with the 3-glucoside positioned at the -1 subsite (data not shown). This explains why hydrolysis at the 4'-position precedes hydrolysis of glucose bound at the 3-position in the double glucoside [17].

The *Tn*Bgl1A aglycone +1 subsite is formed by the hydrophilic residues (N171, H178, N220) and mainly by aromatic and hydrophobic residues (W33, F36, W120, V167, V171, W322, A405, and F412). A comparison with structure determined plant enzymes from GH1 (*Oryza sativa japonica*, *Zea mays) *showed these enzymes to also display aromatic and hydrophobic residues at this subsite but generally with longer hydrophobic residue side chains at the corresponding positions [31].

**Figure 3:**
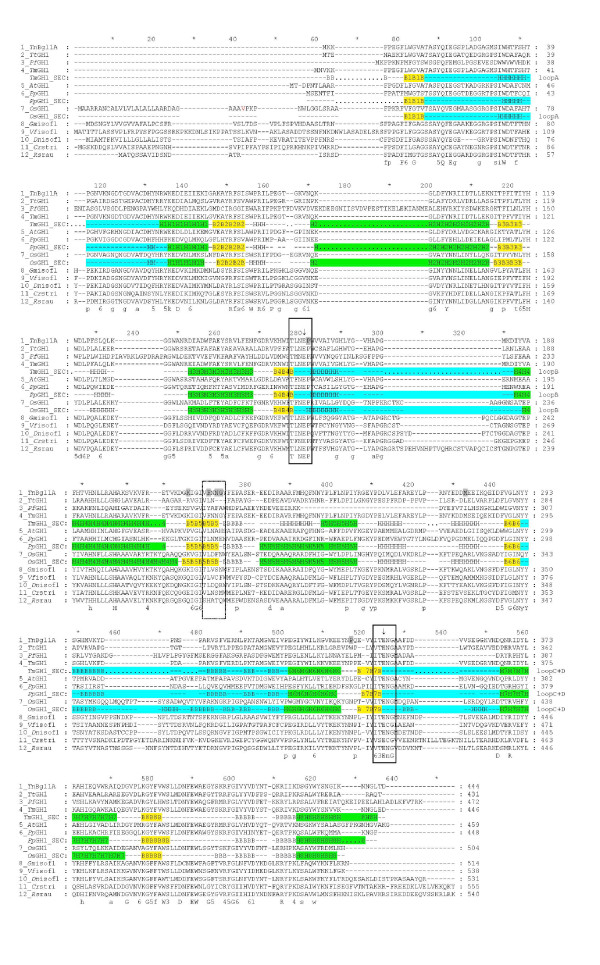
**Multiple sequence alignment of GH1 representatives**. A multiple sequence alignment of β-glucosidases and flavonoid glucosidases from GH1. *Tn*Bgl1A, *Thermotoga neapolitana *Bgl1A (this work); *Tm*GH1, *Thermotoga maritima *BglA (Q08638); *At*GH1, *Agrobacterium tumefaciens *glucosidase (Q7CV27); *Pp*GH1, *Paenibacillus polymyxa *BglB (P22505); OsGH1, *Oryza sativae *(rice) glucosidase (Q42975); *Gm*GH1, *Glycine max *(soy) isoflavonoid glucosidase (AB259819); *Vf*iso, *Viburnum_furcatum *isoflavonoid glucosidase (AB122081)*; Dn*iso, *Dalbergia nigrescens *isoflavonoid glucosidase (AY766303); *Cr*stri, *Catharanthus roseus *strictosidine β-glucosidase (Q9M7N7); *Rs*rau, *Rauvolfia serpentina *raucaffricine β-glucosidase (Q9SPP9). The region selected for mutagenesis is marked by a dashed box, and the two conserved motifs are boxed. The catalytic residues are indicated by arrows. Mutated residues are shaded in grey. Secondary structures are indicated below structure determined enzymes. Helices and strands of the β/α_8_-barrel are numbered and indicated in green and yellow, respectively. The sequence parts corresponding to the four loops (A-D) are indicated in cyan. A consensus sequence is shown in bold below the aligned sequences. Completely conserved resides are shown in upper cases, residues conserved in more than 80% of the sequences are shown in lower case, positions with related residues are indicated by numbers (1 = N, D; 3 = S, T; 4 = K, R; 5 = Y, F, W; 6 = I, L, V, M).

Inspection of residues surrounding the +2 subsite showed a non-conserved region at the "floor" of the active site, more precisely at the end of β-strand 5 (F219, N220, N221, G222, Y223, F224) (Figure 2C). The nonconserved nature of these residues were shown by analysing a multiple sequence alignment as well as by superimposition of known structures. Five more hydrophobic interactions including two aromatic residues (W166, I170, V171, V176, and F310) were also found. Superimposition of 3D structures of GH1 enzymes with varying substrate specificities (of different origin and thermostability such as *Pyroccoccus horikoshii *OT3 (PDB code, 1VFF), *Paenibacillus polymyxa *(PDB code 2Z1S), *Homo sapiens *(PDB code 2JFE), *Oryza sativa japonica *(PDB 2RGL) and *Zea mays *(PDB code 1E4N)) confirmed the variable area of β-strand 5 in the vicinity of the substrate pocket binding as well as variability in the loops. In general, thermophilic β-glucosidases presented shorter loops and more compact overall structures compared to plant counterparts, in line with previous results [32]. The variability at the end of β-strand 5 was also corroborated by the multiple sequence alignment of the *Tn*Bgl1A sequence (Figure 3) with sequences of oligosaccharide, flavonoid-, and isoflavonoid hydrolysing GH1 enzymes as well as GH1 enzymes active on other bulky phenol-containing substrates like *e.g*. the alkaloids strictosidine and raucaffricine (Figure 1C and 1D). The residues at position 219, 221 and 222 were targeted for mutagenesis because of the sequence variation between specificity groups at these sites combined with their location close to the cellotetraose +2 sugar residue. The changes (F219L, N221S, G222Q, G222M) (Figure 2C) were chosen based on residues found in enzymes hydrolysing the bulky phenol-containing substrates (Figure 3). In the case of G222 two mutations were designed, one with an hydrophobic (M) and another one with hydrophilic (Q) residue. Changing a G for M was made to increase hydrophobicity at the entrance of the active site but may exclude water molecules reducing cleavage of the glucosidic linkage in hydrolysis reactions. The G222Q mutation could instead result in substrate interactions via hydrogen bonds (*e.g*. with OH2 and OH3 of a carbohydrate substrate). The F219L and N221S mutations were selected based on residues found at corresponding positions in the enzymes specific for large and bulky substrates, and predicted to generate space for better substrate accommodation.

The residues were mutated in a protocol that introduced a few spontaneous mutations during the amplification procedure. Sequencing of the obtained genes showed that the designed mutations were obtained in all cases, and that one or two spontaneous mutation(s) were present in six clones. The selected clones included four genes with triple mutations: F219L/P165L/M278I, N221S/P165L/M278I, G222Q/P165L/M278I, G222Q/V203M/K214R, two with double mutations: F219L/K214R, N221S/P342L, and two with the single mutation G222M and N221S. The spontaneous mutations (totally five residues) in principle involved conserved changes (replacing a hydrophobic residue with another hydrophobic residue in two cases (V203M, M278I), replacing proline with a hydrophobic residue in two cases (P165L, P342L), and a charged basic residue in one case (K214R). Four of the five spontaneous mutations were located at the surface of the enzyme, opposite the active site. Replacement of proline with leucine at position 165 (P165L) located next to the catalytic acid/base was found in three of the four triple mutants, along with a surface located hydrophobic residue mutation (M278I).

### Expression and purification

**Figure 4:**
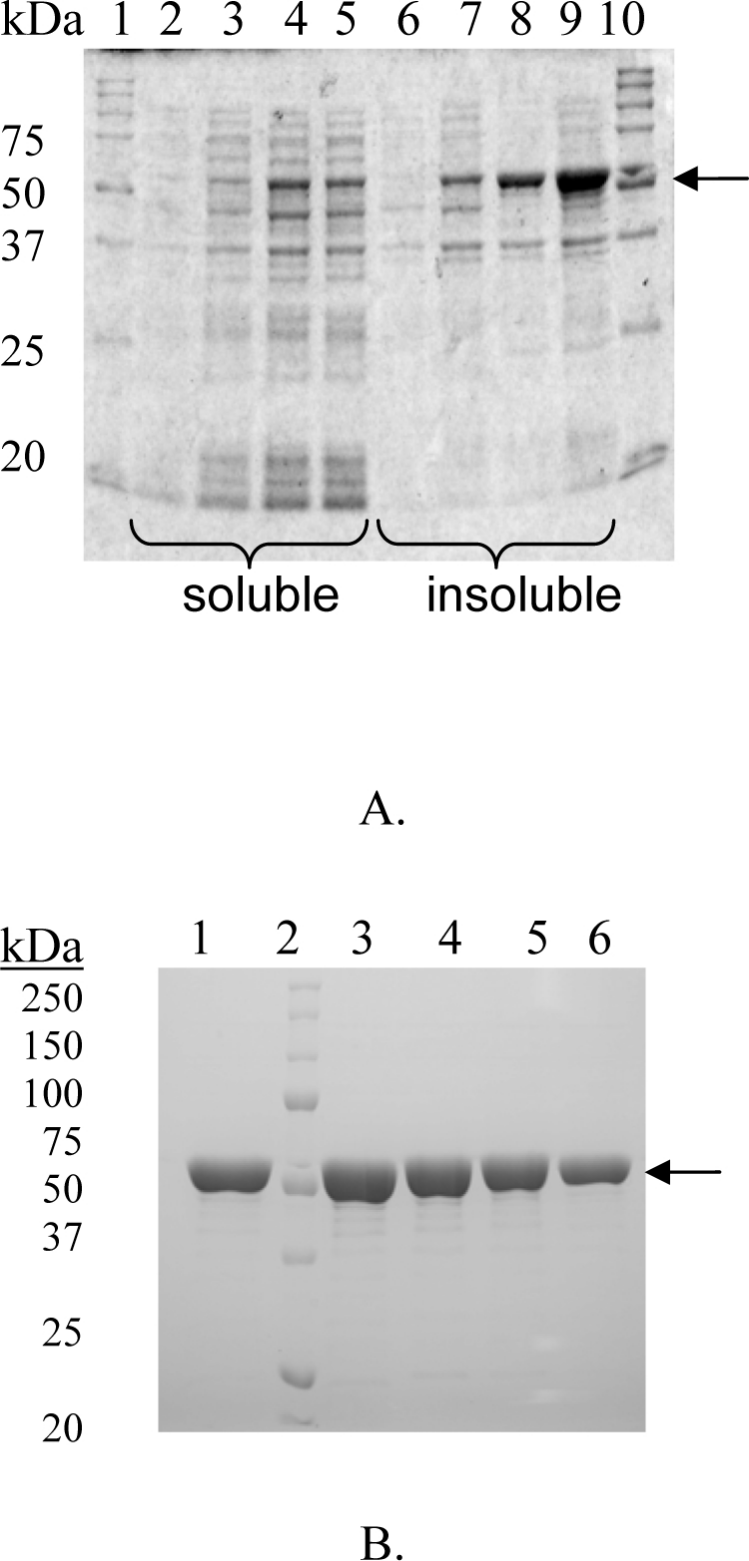
**SDS-PAGE analysis**. Expression of wild-type *Tn*Bgl1A (**A**.). Lane 1, MW standard; lane 2, soluble fraction at induction; lane 3; soluble fraction 1 h; lane 4, soluble fraction 2 h; lane 5, soluble fraction 3 h, lane 6, insoluble fraction 0 h; lane 7, insoluble fraction 1 h; lane 8, insoluble fraction 2 h; lane 9, insoluble fraction 3 h; lane 10, MW standard. *Tn*Bgl1A and some selected mutants purified by IMAC (**B**.). Lane 1, *Tn*Bgl1A; lane 2, molecular weight marker; lane 3, *Tn*Bgl1A-N221S/P342L; lane 4, *Tn*Bgl1A-F219L/K214R; lane 5, *Tn*Bgl1A-G222Q/V203M/K214R and lane 6, *Tn*Bgl1A-G222M.

The wild-type and mutated variants of *T. neapolitana *β-glucosidase 1A were produced in *Escherichia coli *Tuner(DE3) as described by Turner et al [3]. The expression level was analysed by SDS-PAGE and showed all enzymes to have very similar production patterns (Figure 4A), leaving less than half of the produced protein in a soluble active form, despite use of inducer tuning (reducing the IPTG concentration from 1 to 0.1 mM in a lac-permease deficient strain). All mutated enzymes were screened for activity and found to hydrolyse *p*NPGlc (data not shown). Purification was accomplished by a two step protocol, including a heat treatment (70°C, 30 min) followed by immobilised metal ion affinity chromatography (IMAC) utilizing the C-terminal His-tag, which yielded a purity at or above 90% in all cases (Figure 4B).

### Thermostability

**Figure 5:**
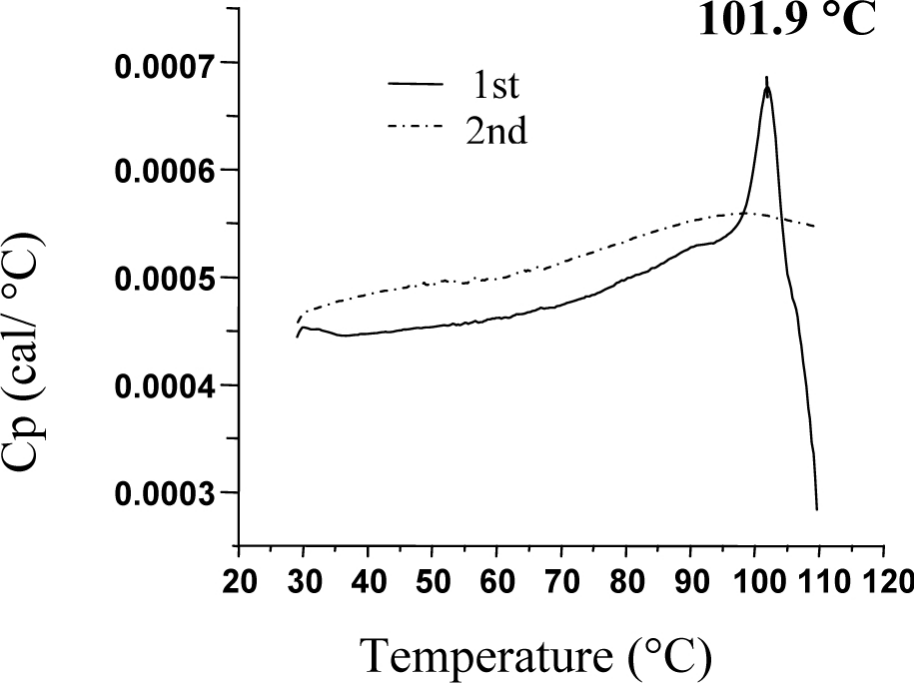
**DSC analysis**. Representative thermogram of wild-type *Tn*Bgl1A (1 mg mL^-1 ^in 20 mM citrate phosphate buffer, pH 5.6).

Thermostability of all enzyme variants was evaluated by differential scanning calorimetry (DSC). A single transition peak was observed in all cases, which during unfolding resulted in aggregation (also manually observable in the sample after scanning). A repeated scan confirmed the denaturation to be irreversible in all cases (Figure 5). All enzyme variants kept unfolding temperatures above 90°C making them suitable as biocatalysts in applications requiring high thermostability. As expected, thermostability decreased with increasing number of mutations, and the triple mutants showed a decrease in the apparent unfolding temperature (ΔT_m_) ranging from 5 - 10°C (Table 2). The single and double mutants did not change unfolding temperature to any large extent.

**Table 2:** The melting temperatures of the different expressed constructs measured by differential scanning calorimetry.

Enzyme	T_m _(°C)	ΔT_m _(°C)
wild-type	101.9	
N221S/P342L	100.5	-1.4
N221S/P165L/M278I	95.5	-6.4
F219L/K214R	101.8	-0.1
F219L/P165L/M278I	93.4	-8.5
G222Q/V203M/K214R	96.8	-5.1
G222Q/P165L/M278I	94.8	-7.3
G222M	101.2	-0.7

### Kinetic parameters in *p*NPGlc hydrolysis

The kinetic parameters for hydrolysis were monitored using the model substrate *p*NPGlc at pH 5.6 (Table 3). The pH was set based on previously published data taking into account the pH-range for the highest k_cat_/K_M _determined for the homologous *T. maritima *enzyme (pH optimum 5.8 ± 0.2) [24] combined with the three point screening of *Tn*Bgl1A (pH 3, 5 and 7, showing highest activity at pH 5) for Q4' hydrolysis [3]. The turnover number as well as the K_M _values showed changes in some variants. The mutation at position 222 (in G222M, G222Q/P165L/M278I, and G222Q/V203M/K214R) resulted in a significant reduction in the turnover number (k_cat_) both for the single G222M mutant and the triple mutants including the change G222Q as compared to the *wt*. Only a minor reduction in catalytic efficiency (k_cat_/K_M_), was however seen, as the variations in K_M _in most cases counteracted the change in turnover. The triple mutant F219L/P165L/M278I showed a similar reduction in turnover, but this is likely an effect of added mutations, as the double mutant carrying the F219L change (F219L/K214R) showed parameters more similar to the wild type. Variants carrying P165L, located next to the catalytic acid/base, showed in all cases a lower turnover than other variants carrying changes in either F219, N221 or G222, but lacking the change at position 165 (Table 3). All three mutants carrying the N221S change instead showed an increased turnover (3.5 ×, 2.4 × and 2.2 ×). The increase in turnover was however combined with a significant increase in K_M _for all three mutants (N221S/P342L, N221S/P165L/M278I and N221S) which displayed 3.7 ×, 2.7 × and 1.8 × increases in K_M_, respectively. As the increased K_M _counteracted the increase in turnover only a minor change in catalytic efficiency was observed. The relatively more pronounced effect on the K_M _however raised an interest for trying these variants in deglycosylation reactions of the quercetin glucosides, as glucose at the 3-position generally seems more difficult to hydrolyse with the GH1 enzymes (see below).

**Table 3:** Michaelis-Menten constants.

Enzyme	K_M _(mM)	k_cat _(s^-1^)	k_cat_/K_M _(s^-1 ^mM^-1^)
*p*NPGlc-hydrolysis			

*Wild-type*	0. 24 ± .04	485 ± 31	2000

N221S	0.43 ± 0.11	1170 ± 152	2720
N221S/P342L	0.89 ±0.19	1710 ± 237	1910
N221S/P165L/M278I	0.66 ±0.18	1070 ± 171	1630
F219L/K214R	0.24 ±0.04	594 ± 36	2510
F219L/P165L/M278I	0.18 ±0.006	253 ± 3	1440
G222Q/V203M/K214R	0.10 ± .008	154 ± 3	1470
G222Q/P165L/M278I	0.27 ± .05	181 ± 15	678
G222M	0.17 ±0.03	254 ± 16	1470

Q4'-hydrolysis*			

*Wild type*	0.06 ±0.03	7.2 ± 1.2	122
N221S/P342L	0.02 ±0.015	6.2 ± 0.95	281

Q3-hydrolysis*			

*Wild type*	0.13 ±0.06	7.2 ± 1.8	55
N221S/P342L	0.05 ±0.02	7.1 ± 1.1	154

*Data from Lindahl et al [17].

For *p*NPGlc hydrolysis measurements were made at pH 5.6, 80°C, and for the quercetin glucoside hydrolysis (*wt *and one mutant) at pH 5.0, 90°C.

Previous work by Lindahl et al, and Yernool et al [17,18], have shown that glucose is acting as an activator in the *wt *enzyme. To assure that this effect is maintained after mutation, the kinetics of the *wt *and N221S/P342L variant in *p*NPGlc hydrolysis were determined in presence of 10 mM glucose. This resulted in an increase of the turnover (k_cat_= 784 s^-1 ^*wt*, k_cat_= 2310 s^-1 ^N221S/P342L) but no significant change in the K_M _value (K_M _= 0.25 ± 0.02 *wt *, K_M _= 0.87 ± 0.08 N221S/P342L) leading to an increased catalytic efficiency in presence of glucose (k_cat_/K_M _= 3150 *wt*, k_cat_/K_M _= 2650 N221S/P342L). The activating effect of glucose is hence maintained to the same extent in the mutated enzyme, and no product inhibition upon glucose release is expected.

### Quercetin-glucoside hydrolysis

**Figure 6:**
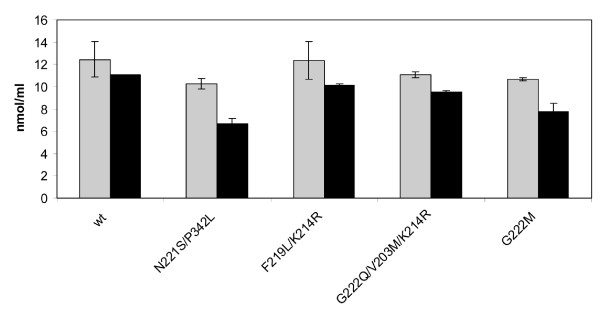
**HPLC analysis**. Analysis of quercetin-3-glucoside (Q3). Grey bars represent the Q3 concentration at time 0 min, and black bars remaining Q3 concentration after 5 min incubation with the enzyme.

**Figure 7:**
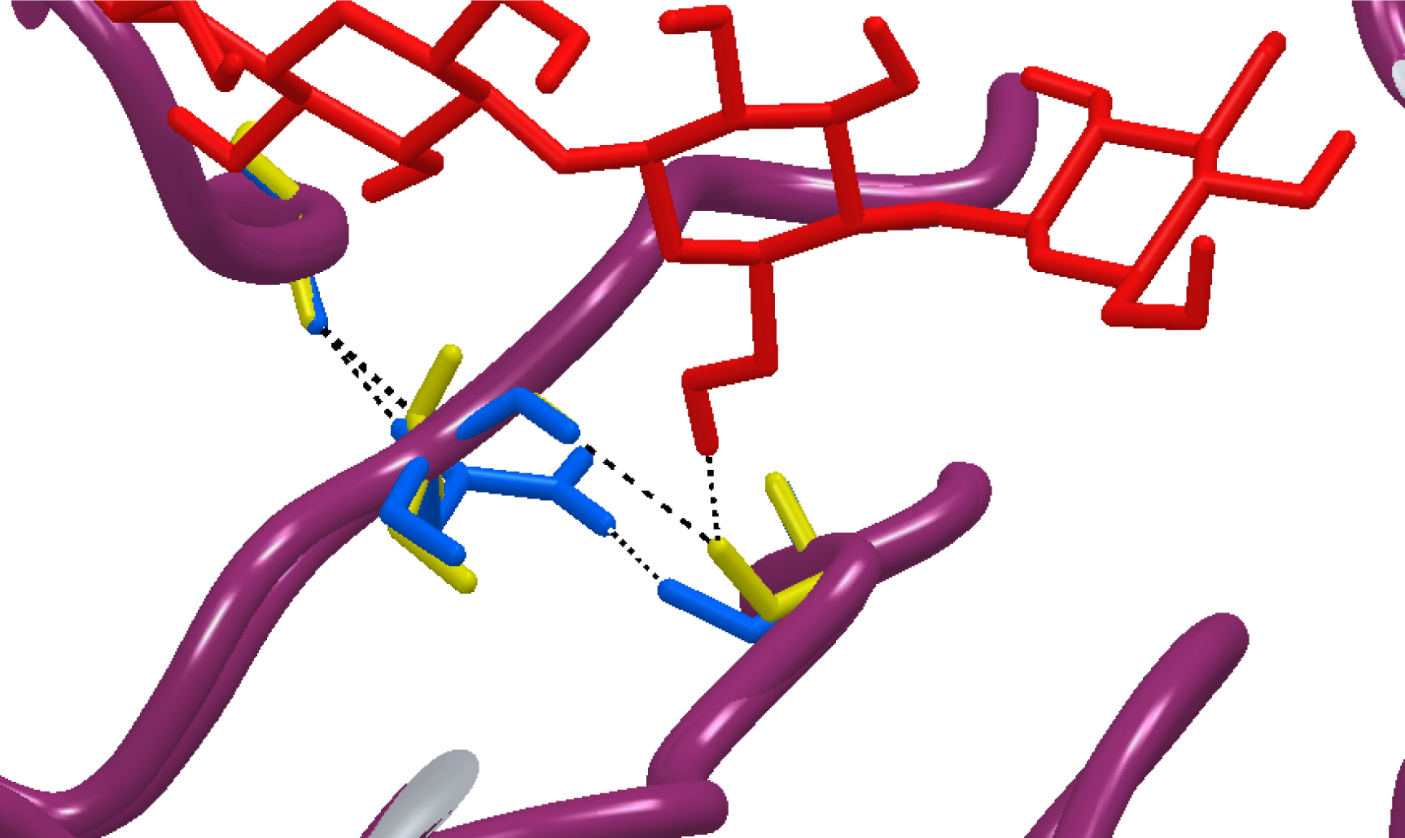
**Interactions around position 221**. The *Tn*Bgl1A homology model with a cellotetraose ligand with and without residue N221 (blue) mutated to S (yellow). The S mutation allows for recognition of the 6-hydroxyl group of the glucose in the +1 subsite and of the 5-hydroxylgroup of quercetin, by hydrogen bonding to the backbone carbonyl oxygen of residue N291.

Although hydrolysis of different quercetin-glucosides by enzymes from GH1 has been reported [10], hydrolysis of the Q3 glucoside appears to be more unusual. In the case of *Tn*Bgl1A *wt*, it has been shown that hydrolysis of Q3 is possible but slow [3,17]. This motivated screening of obtained mutants in Q3 hydrolysis, to monitor improvements in the hydrolysis of this substrate using a fixed concentration of enzyme and substrate (Figure 6). Of the positions selected at the +2 site the mutation N221S led to the highest increase in conversion (from 11 to 35%) of Q3 to Q (Figure 7). Repeated trials with the N221S single mutant, showed that the second mutation P342L in the double mutant, had no major role in this increase (data not shown). The G222M mutation also led to increased Q3-conversion (27%). These improvements may be a result of improved substrate accomodation, and indeed the modelling of Q3 in the N221S variant showed that an additional hydrogen bonding to the substrate (5-OH on the quercetin backbone) can occur via the backbone carbonyl of N291 as a consequence of interaction changes caused by the mutation (Figure 7). Increased hydrophobicity could improve interactions between the substrate and enzyme, and may be the case for G222M which likely has its sidechain pointing into the catalytic cleft. For Q4' all enzyme variants completely hydrolysed the substrate within the reaction time of 10 min.

The (N221S/P342L) mutant resulted in the highest conversion of Q3 and was selected for determination of the kinetic parameters for conversion of the Q4' and Q3 glucosides to Q (Table 3, [17]). Largest improvement was due to a decrease in K_M _using both Q4'and Q3, and this was especially pronounced using the Q3 substrate (Table 3), which can be explained by the added substrate interaction (Figure 7).

## Discussion

The thermostable β-glucosidase (*Tn*Bgl1A) from *T. neapolitana *has been used as a biocatalyst for conversion of quercetin-glucosides to quercetin [3] extracted using a hot water extraction method, shown to be beneficial from an environmental perspective [17]. It was shown that the enzyme completely converted the Q4' species to quercetin, and that also the glucoside connected to the 3-position of the flavonoid backbone (Q3) was converted, although with lower efficiency. This shows that the accessibility to the active site differs between enzymes in GH1, as previous work on flavonoid hydrolysing enzymes in GH1, have shown that enzymes capable of converting the 4'-glucoside (e.g. human β-glucosidase (*h*CBG)) are not capable of hydrolysing the flavonoid 3-glucosides [10,33]. The *Tn*Bgl1A enzyme is thus an interesting target to study by molecular modelling, as well as by site-directed mutagenesis to analyse how changes of amino acids affect the kinetic parameters and the conversion of the quercetin-3-glucoside.

**Figure 8:**
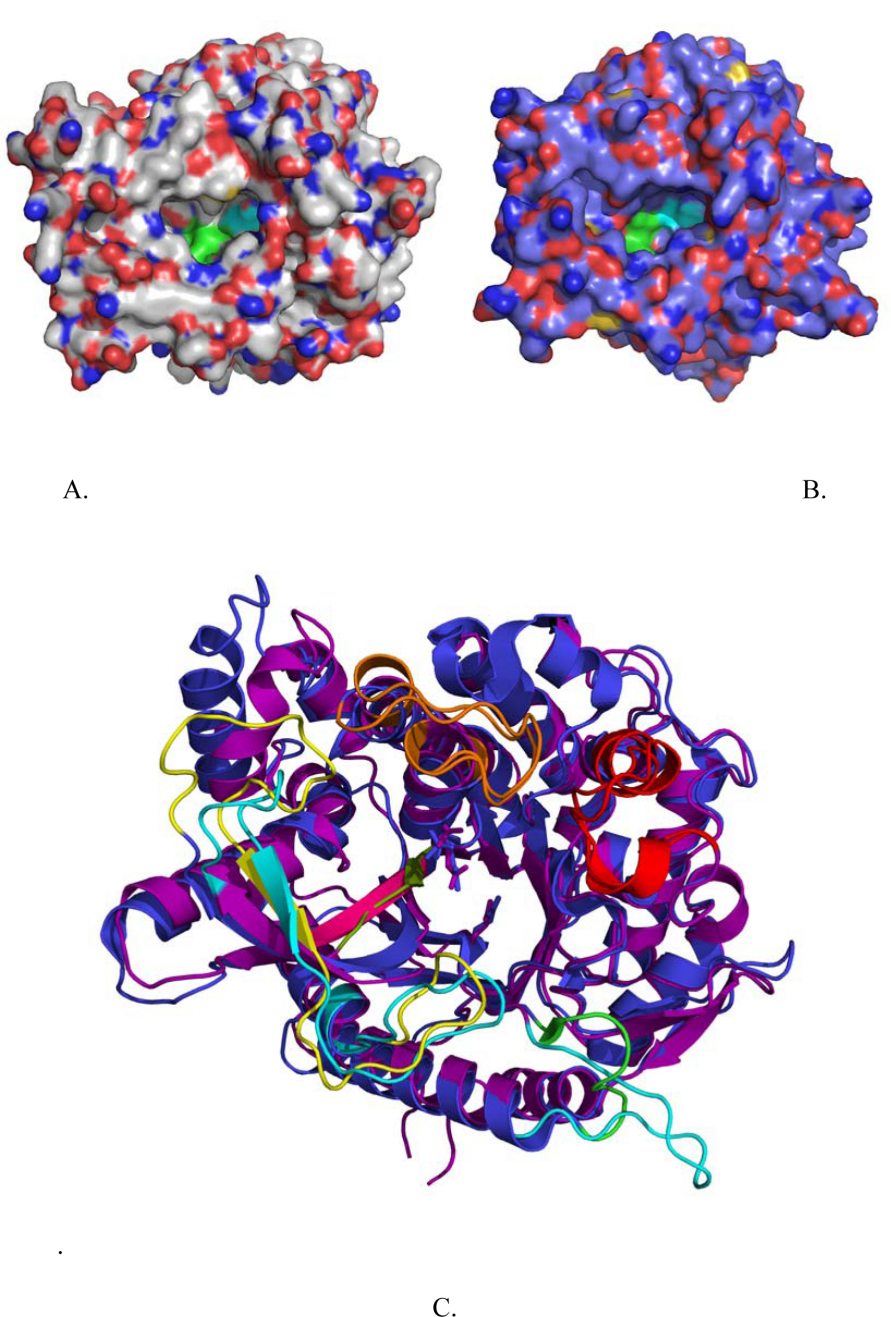
**Structural comparison of *h*CBG and *Tn*Bgl1A**. Panel A and B show a surface view of *h*CBG (**A**.) and *Tn*Bgl1A (**B**.). The smaller entrance at the *h*CBG active site and wider entrance of the *Tn*Bgl1A active site are clearly visible. The overall similarity of the structures is shown by superimposition (**C**.) of the *Tn*Bgl1A (purple) and *h*CBG (blue) structures. The active site residues are shown as sticks. The fours loops surrounding the active site are indicated. Loops A (red), and B (orange) do not show differences but loop C (yellow in *h*CBG, cyan in *Tn*Bgl1A) and loop D (green in *h*CBG, cyan in *Tn*Bgl1A) presented big differences. Loop C, around the active site entrance, seems bigger in *h*CBG compared to *Tn*Bgl1A. Loop D on the other hand is long for *Tn*Bgl1A compared to the small and compact loop in *h*CBG. The β-strand close to the active site area (pink in *Tn*BglA, green in *h*CBG) was chosen for mutagenesis.

Interactions with inhibitors in the -1 subsite of the homologous *T. maritima *enzyme have been extensively studied [27,28,34,35], and have shown this site to be important for the selection of the sugar to be hydrolysed. In a study on a *Sulfolobus solfataricus *representative of GH1 by Corbett et al, [36] it was for example shown that mutagenesis of substrate interacting residues in the -1 site led to a shift in affinity towards xylose, or from glucose to mannose, dependent on the residue chosen. Here we are instead focusing on interactions closer to the entrance of the active site, and close to the +2 site, which has not been targeted to the same extent. Comparison of the structure of human CBG, which cannot hydrolyse the flavonoid 3-glucoside [10,33] with the model of *Tn*Bgl1A, shows a difference in the shape of the active site (Figure 8A and 8B). It can be clearly seen that *h*CBG forms a regular oval shaped pocket, while the corresponding pocket in *Tn*Bgl1A (as well as in *Tm*BglA used as template for modelling) has a wider conformation, which may facilitate accomodation of the 3-linked substrate. The active site entrance in GH1 is formed by four extended loops [30]. These loops (termed loop A-D) have been defined as being responsible for the overall shape of the aglycone binding pocket, and differences in the conformation of one or more of these loops would likely change the overall shape of the entrance [33]. In *h*CBG, loops B (residue 173-187, *h*CBG numbering) and D (residue 378-385, *h*CBG numbering) are short, which is claimed to result in a small entrance to the pocket. Comparison with *Tn*Bgl1A show that loop length of loop B is the same as in *h*CBG, and despite low sequence similarity in this area both loop A and loop B superimpose very well between the two enzymes. Loop D is however longer in *Tn*Bgl1A, in accordance with the suggestion that this may contribute to a wider active site entrance of this enzyme (Figure 8B and 8C). However, parts of loop C do not superimpose between the two enzymes and in *Tn*Bgl1A this loop is significantly shorter than in the human enzyme (Figure 8C). It is actually close to this shorter loop C that we see a significant widening of the *Tn*Bgl1A active site compared to *h*CBG, indicating that the longer loop in the human enzyme is closing the active site entrance. Moreover, the mutations introduced at the +2 site are located close to loop C in the structure and may aid in a further widening of the active site entrance simplifying binding of Q3.

In addition to interactions at the +2 subsite, the modelling of Q3 into the active site of *Tn*Bgl1A showed that +1 subsite binding was also affected as a consequence of changes in the interactions between residues. The side chain of residue N221 selected here, which upon mutagenesis led to a decrease in K_M _for Q3 as well as increased catalytic efficiency, is not pointing towards the catalytic cleft in our model. A close look at the binding interactions of N221 in presence of Q3 shows that this residue is interacting with residues in neighbouring strands of the protein (the backbone carbonyls of Y246 and N291) (Figure 7), and N221 may thus have importance for the shape in that part of the active site. The introduced S221 leads to loss of the interaction with N291, but also a change in the position of the backbone carbonyl of the residue allowing hydrogen bonding to the 5-OH of Q3 (located in a position corresponding to the +1 sub site). Such a bond would explain the observed decrease in K_M_. Calculation of the ΔΔG (= -RT ln([k_cat_/K_M _]_wt_/[k_cat_/K_M _]_mut_)) for Q3 corresponded to a free energy change of 3 kJ/mol. A hydrogen bond interaction to an uncharged amino acid is in the range 2-6 kJ/mol [37] showing that the change in K_M _is likely the result of an affinity increase. Changes in turnover are more difficult to explain, and may be caused by conformational changes caused by indirect changes in interactions between residues. Other explanations include changes in the position of the side chain of the neighbouring residue (N220, interacting with the +1 site in *Tn*Bgl1A) pointing into the catalytic site. A similar position of the sidechain is found for the corresponding residue in the homologous human glucosidase (F225 in the *h*CBG structure). The *h*CBG residue F225 was shown by mutagenesis to affect the aglycone specificity [10]. Mutagenesis of the neighboring residue (N221) in *Tn*Bgl1A may change the position of N220 in the active site, or lead to changes in the local environment that promotes an affinity change. Mutation of F219 (also with the side chain located away from the catalytic cleft), preceding N220, did however not lead to any corresponding or significant changes in affinity or turnover.

All spontaneous mutations, except one, involved residues located at the surface of the enzyme. Only P165L of the spontaneous mutations, is located in the active site next to the catalytic acid/base. This change from proline to leucine should introduce more flexibility. The effect of this change on the activity is, based on the activity data, however not completely clear. It may lead to a minor decrease in activity compared to clones where this mutation is lacking, but its effect appears to be small. The additional surface located mutations, appear to mostly affect stability (slight destabilisation), but it is only in triple mutants that the transition temperature is affected to any large extent (> 5°C).

## Conclusions

In conclusion, the mutation study done in this work shows that relatively small residue variations in the enzyme, made at or close to the +2 site, may modify the interactions in the active site, leading to increased substrate interactions as well as conformational changes that allow increased hydrolysis of a sterically differently attached glucose on the quercetin backbone. In addition, effects on the turnover of the introduced mutations were often counteracted by a change in K_M_, leading to smaller differences in catalytic efficiency, than in the separated K_M _and k_cat _parameters.

## List of abbreviations

DSC: differential scanning calorimetry; G2F: 2-deoxy-2-fluoro-beta-D-glucoside; GH: Glycoside Hydrolase; hCBG: human cytosolic beta-glucosidase; HPLC: high pressure liquid chromatography; K_M_: The Michaelis constant; k_cat_: turnover number; PCR: polymerase chain reaction; *p*NPGlc: para-nitrophenyl-beta-D-glucopyranoside; *p*NP: para-nitrophenol; *Tn*Bgl1A: *Thermotoga neapolitana *beta-glucosidase A from Glycoside hydrolase family 1; Q: quercetin; Q3: quercetin-3-glucoside; Q4': quercetin-4'-glucoside; SDS-PAGE: sodium dodecyl sulfate polyacrylamide gel electrophoresis; 3D: three dimensional

## Authors' contributions

SK: Took part in the construction and sequence analysis of mutants, took part in enzyme production, purification and kinetics, and in DSC-data collection. Took part in writing the manuscript and commenting on the manuscript.

TP: Took part in constructing mutants. Did the homology modelling and structure analysis together with AS, Took part in writing the manuscript and commenting on the manuscript.

MM: Took part in the construction and sequence analysis of mutants, in the enzyme production and purification, and in DSC-data collection. Took part in writing the manuscript.

SL: Did the hydrolysis and HPLC-analysis of quercetin and quercetin-3-glucosides, Took part in writing and commenting on the manuscript.

AS: Did the major part of the modelling (together with TP), and wrote some parts related to this work in the manuscript.

CT: Took part in the interpretation of HPLC-data. Took part in writing and commenting on the manuscript

ENK: Selected enzyme and mutation strategy. Took part in the bioinformatics analysis and the DSC-data collection. Took part in overall analysis and interpretation of results. Did the overall layout of manuscript, took part in writing and commenting on the manuscript.

All authors read and approved the final manuscript.
